# Gemcitabine plus nab-paclitaxel for locally advanced or borderline resectable pancreatic cancer

**DOI:** 10.1038/s41598-019-52486-x

**Published:** 2019-11-07

**Authors:** Akiko Tsujimoto, Kentaro Sudo, Kazuyoshi Nakamura, Emiri Kita, Ryusuke Hara, Wataru Takayama, Hiroshi Ishii, Taketo Yamaguchi

**Affiliations:** 1 Division of Gastroenterology, Chiba Cancer Centre, Chiba, Japan; 2 Division of Radiation Oncology, Chiba Cancer Centre, Chiba, Japan; 3 Division of Hepatobiliary and Pancreatic Surgery, Chiba Cancer Centre, Chiba, Japan

**Keywords:** Pancreatic cancer, Chemotherapy

## Abstract

Overall survival in a phase III study for metastatic pancreatic cancer has significantly improved with gemcitabine (GEM) plus nab-paclitaxel. However, to date, there is limited data on the efficacy and safety of its use for patients with locally advanced (LA) or borderline resectable pancreatic cancer (BRPC). Here, we investigated the efficacy and safety of first-line GEM plus nab-paclitaxel for LA or BRPC. We retrospectively analysed consecutive patients with pathologically confirmed, untreated LA or BRPC who started receiving first-line GEM plus nab-paclitaxel. A total of 30 patients (LA, n = 22; BRPC, n = 8) were analysed. Twelve patients (40%) without distant metastasis received additional chemoradiotherapy using S-1. Laparotomy was performed on 8 patients and 6 (20%; LA, n = 3; BR, n = 3) achieved R0 resection. Objective response rate was 44.8%. For all patients, median progression-free survival and overall survival were 14.8 and 29.9 months, respectively. Median overall survival for LA was 24.1 months with a 2-year survival rate of 50.8%. The most frequently observed grade 3 or 4 toxicities were neutropenia (73%) and biliary infection (13%). First-line GEM plus nab-paclitaxel was well-tolerated and feasible with an encouraging survival for LA or BRPC.

## Introduction

Locally advanced (LA) or borderline resectable pancreatic cancer (BRPC) are subsets of PC which account for about 30–40% of all patients^1,2^. Patterns of disease progression and survival outcomes differ from those with metastatic disease^1,3^. To date, systemic chemotherapy, combined or not with chemoradiotherapy (CRT), represents the standard treatment for LAPC^4^. In addition, neoadjuvant therapy followed by surgical resection is the preferred option in BRPC, despite a lack of high-level evidence^5^.

However, it remains unclear what is the best first-line therapy in LA or BRPC patients. Up to the early 2010s, CRT or gemcitabine (GEM)-based systemic chemotherapy were the mainstay in the treatment of LAPC. However, it has been shown that those conventional therapies provided limited survival benefits, having a reported median survival of 8–17 months^6–12^. Consequently, it is urgent to develop more effective therapies.

In a large-scale, international phase III study for metastatic pancreatic cancer (MPACT), it has been shown that GEM plus nab-paclitaxel significantly improved both overall survival and progression-free survival (PFS) compared to GEM alone^13^. Additionally, an improved radiographic response was observed (23% vs. 7%, P < 0.001). As stated in the National Comprehensive Cancer Network (NCCN) guidelines of pancreatic adenocarcinoma, GEM plus nab-paclitaxel is currently accepted as an option in the management of LA or BRPC^5^. However, to date, there is limited data available on the safety and efficacy of its use for this population. To address this issue, we retrospectively investigated the efficacy and safety of first-line GEM plus nab-paclitaxel for LA or BRPC in the present study.

## Results

### Patients

**Figure 1:**
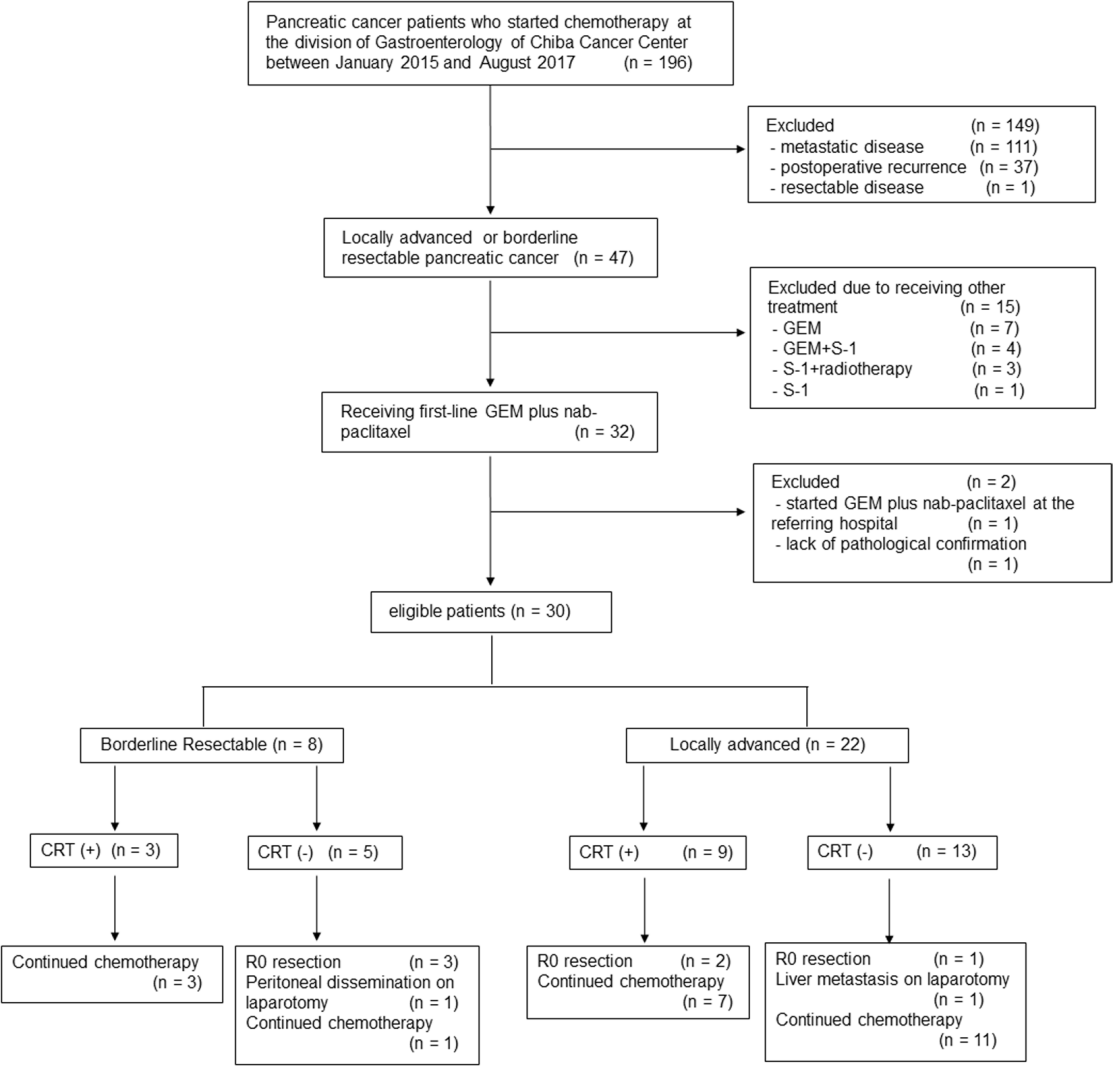
Consort Diagram.

A total of 196 patients with pancreatic cancer started chemotherapy at the division of Gastroenterology of Chiba Cancer Centre between January 2015 and August 2017 (Fig. 1). Of the 196 patients, 47 patients had LA or BR disease at the initiation of chemotherapy. Of these, 17 patients were excluded due to receiving other treatment (n = 15), starting GEM plus nab-paclitaxel at the referring hospital (n = 1) or lack of pathologic confirmation (n = 1). Thus, 30 patients with LA unresectable (n = 22) and BRPC (n = 8) were eligible for the study (Fig. 1). Table 1 shows the baseline characteristics of the study population. The median age was 67 years. All patients had a good performance status (PS) of 0 or 1. The primary tumour was located in the head of the pancreas in 18 patients (60%) and it was in the body/tail in 12 (40%). Biliary drainage was required prior to treatment initiation in 16 patients (53%). Specifically, 14 patients underwent endoscopic biliary stent placement and 2 underwent choledochojejunostomy. The median tumour size was 44 mm. Median serum CA19–9 concentration was 160 U/mL.

**Table 1:** Patient characteristics. Abbreviations: ECOG PS, Eastern Cooperative Oncology Group performance status; BR, borderline resectable; LA, locally advanced; SMA, superior mesenteric artery; CA, celiac axis; HA, hepatic artery; SMV, superior mesenteric vein; PV, portal vein.

	n or median (range)
n	30
**Gender**	
Male	14
Female	16
Median Age, years (range)	67 (47–75)
**ECOG PS**	
0	16
1	14
**Site of primary tumour**	
Head	18
Body or tail	12
**Biliary drainage**	
Stent	14
Bypass	2
Median tumour size, mm (range)	44 (25–88)
**Resectability**	
BR LA	8 22
**Vessel invasion**, (**%**)	
SMA	24 (80%)
CA	15 (50%)
HA	18 (60%)
SMV/PV	25 (83.3%)
CA19–9 (U/mL), median [range]	160 [<2–44074]

### Treatment

Figure 1 summarizes the treatment for all the patients. The initial doses of GEM or nab-paclitaxel were reduced by more than 10% in 4 patients (13.3%) because of repeated biliary tract infection (n = 1), patient’s wish (n = 1), co-existing disease (n = 1) or physician’s discretion (n = 1). The remaining patients (n = 26) started GEM plus nab-paclitaxel at standard dosage. Treatment was terminated due to adverse events (AEs) in 2 patients. The median duration for first-line GEM plus nab-paclitaxel was 5.4 months (range: 1.4–25.8 months). In total, 90% (n = 27) of the patients required dose modification (dose reduction, n = 12; treatment schedule modification, n = 3; both, n = 12) owing to the development of toxicity. Forty-three percent of the patients had dose reduction of GEM and 80% had dose reduction of nab-paclitaxel. Of the 30 patients, 12 (40%) received S-1-based CRT after the first-line GEM plus nab-paclitaxel, followed by maintenance chemotherapy using GEM plus nab-paclitaxel (n = 4), S-1 alone (n = 5) or GEM plus S-1 (n = 1). The median duration from the start of GEM plus nab-paclitaxel to CRT initiation was 6 months (range, 2.8–8.5 months). The median duration from the completion of CRT to the start of maintenance chemotherapy was 2.5 weeks (range, 1.1–4 weeks).

### Safety

All the patients (n = 30) were evaluable for AEs. Table 2 shows Grade 3 or 4 AEs observed during the first-line GEM plus nab-paclitaxel. The most common Grade 3 or 4 AE were neutropenia (73% of the patients), followed by biliary infection (13% of patients). All the patients who developed biliary infection had pancreatic head tumour and had undergone biliary stent placement. Among these patients, one patient developed acute cholecystitis during chemotherapy with GEM plus nab-paclitaxel. Repeated biliary tract infection after biliary stent placement was the main cause of acute cholecystitis. The patient required percutaneous gallbladder drainage followed by elective cholecystectomy. Biliary tract infection in other patients was mainly induced by stent occlusion, and this was managed by stent replacement and antibiotic treatment.

**Table 2:** Grade 3 or 4 adverse events.

	n (%)
White blood cell decreased	5 (17%)
Neutrophil count decreased	22 (73%)
Febrile neutropenia	0
Anaemia	2 (7%)
Anorexia	1 (3%)
Nausea	1 (3%)
Biliary tract infection	4 (13%)
Pneumonitis	1 (3%)
Lung infection	1 (3%)
Haematuria	1 (3%)
Fatigue	0
Peripheral neuropathy	0
Diarrhoea	0

Furthermore, two patients had additional AEs. One patient developed interstitial lung disease, likely induced by GEM, the other developed pneumocystis pneumonia. Both patients recovered with conservative therapy. We did not observe cases of febrile neutropenia or treatment-related death.

### Response

Twenty-nine patients were evaluable for radiographic response. One patient was excluded from the evaluation of objective response due to the lack of a measurable lesion. During chemotherapy with GEM plus nab-paclitaxel, 13 patients (44.8%) achieved a partial response, 15 (51.7%) had stable disease and 1 (3.4%) developed disease progression. Among the 15 patients with stable disease, 2 showed a partial response after additional CRT. Therefore, 15 patients (51.7%) achieved a partial response following multimodal treatment using GEM plus nab-paclitaxel.

Overall, 8 patients (26.7%; BR, n = 4; LA, n = 4) underwent laparotomy with a curative intent (Fig. 1). Three patients with BRPC received GEM plus nab-paclitaxel as neoadjuvant therapy, and they underwent either pancreatoduodenectomy (n = 2) or distal pancreatectomy (n = 1) at 2.2–5.6 months after treatment initiation. They all achieved R0 resection. Further, one patient with BRPC who showed partial response after receiving first-line GEM plus nab-paclitaxel therapy underwent laparotomy at 24.4 months after treatment initiation. However, this patient was found to be inoperable owing to peritoneal dissemination. Three patients with LAPC who showed significant radiographic response underwent either pancreatoduodenectomy (n = 2) or distal pancreatectomy (n = 1) at 6.1–10.2 months after treatment initiation. They all achieved R0 resection. Of note, one patient treated with additional CRT showed a pathologic complete response. Another patient with LAPC underwent laparotomy at 3.4 months after treatment initiation, but this patient was found to be inoperable owing to liver metastasis.

### Survival

**Figure 2:**
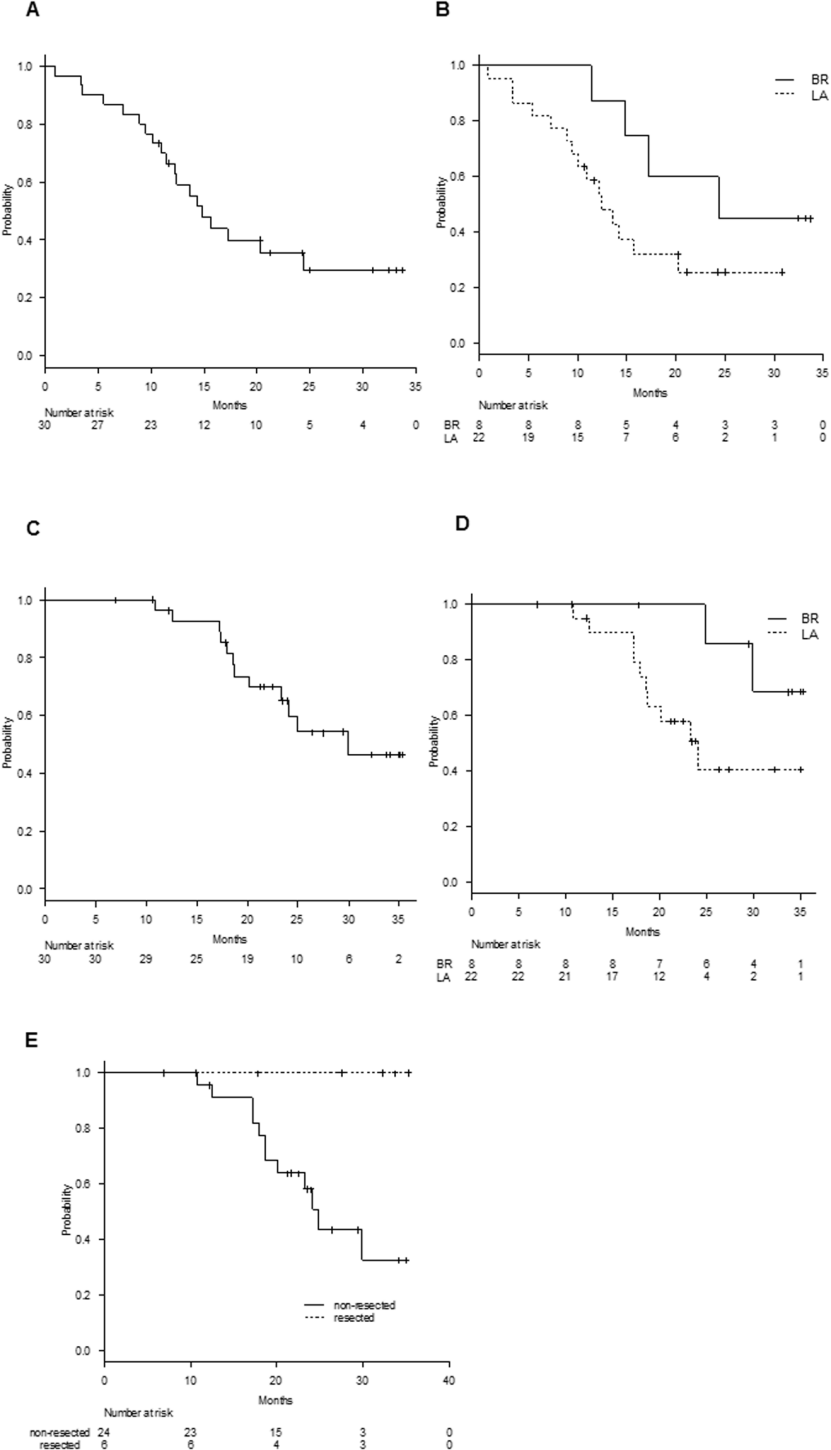
(**A**) Progression-free survival for all patients (n = 30) and (**B**) for LA (n = 22) and BR (n = 8) patients. (**C**) Overall survival curves of all patients (n = 30) and (**D**) LA (n = 22) and BR (n = 8) patients. (**E**) Overall survival for resected (n = 6) and non-resected (n = 24) patients.

The median follow-up length for censored cases was 25.2 months (range: 6.9–35.3 months). Disease progression was observed in 19 patients. The median PFS for all treated patients (n = 30) was 14.8 months (95% CI, 11.4–24.4) (Fig. 2A). Specifically, median PFS for LA (n = 22) and BRPC patients (n = 8) was 12.4 months (95% CI, 8.9–20.3) and 24.4 months (95% CI, 11.4–NA), respectively (Fig. 2B).

For all treated patients (n = 30), the median overall survival was 29.9 months (95% CI, 20.1–NA) with a 2-year survival rate of 65.1% (95% CI, 43.4–80.2%) (Fig. 2C). For LA patients (n = 22), median overall survival and survival rate at 2 years were 24.1 months (95% CI, 17.9–NA) and 50.8% (95% CI, 26.1–71%), respectively (Fig. 2D). The median overall survival of BR patients (n = 8) was not reached (Fig. 2D). The median overall survival for 24 patients who did not undergo pancreatectomy was 24.9 months (95% CI, 18.6–NA) with a 2-year survival rate of 58% (95% CI, 34.4–75.7%) (Fig. 2E).

## Discussion

There is limited data available as to the efficacy and safety of GEM plus nab-paclitaxel in patients with LA or BRPC. Gulhati *et al*. assessed the use of first-line GEM plus nab-paclitaxel for the treatment of localised pancreatic cancer in a large case series (n = 99)^14^. In that study, most of the patients (81%) received biweekly doses of GEM plus nab-paclitaxel. The median survival of all the patients, including those with resectable pancreatic cancer (n = 45), was 18 months, but specific survival data for patients with BR (n = 14) or LAPC (n = 40) have not been presented.

In the present study, we demonstrated that first-line GEM plus nab-paclitaxel was feasible. Additionally, we showed that this line of treatment is associated with an encouraging survival outcome for this population. Median overall survival of patients treated with GEM plus nab-paclitaxel (all patients, 29.9 months; LA patients, 24.1 months) appeared to be better than for patients treated with conventional therapies (e.g. upfront CRT or GEM-based systemic chemotherapy) (8–17 months) (Table 3)^6–12^. Furthermore, median overall survival of 24 patients (80%) treated with non-surgical treatment alone reached 24.9 months.

**Table 3:** Clinical studies of conventional CRT or GEM based chemotherapy for LAPC. Abbreviations: P II, phase II study; P III, phase III study; GEM, gemcitabine; CRT, chemoradiotherapy; RT, radiation therapy; PFS, progression-free survival; TTP, time to progression; MST, median survival time; NA, not available.

Author	design	CRT or systemic chemotherapy	n	Response rate	PFS or TTP (months)	MST (months)	ref.
Ishii	P II	5-FU + RT (50.4 Gy)	20	10%	4.9	10.3	6
Sudo	P II	S-1 + RT (50.4 Gy)	34	41%	8.7	16.8	7
Okusaka	P II	GEM + RT (50.4 Gy)	42	21%	4.4	9.5	8
Loehrer	P III	GEM + RT (50.4 Gy) GEM	34 37	6% 5%	6.0 6.7	11.1 9.2	9
Chauffert	P III	5-FU + Cisplatin + RT (60 Gy) GEM	59 60	NA NA	14%^a^ 32%^a^	8.6 13.0	10
Ishii	P II	GEM	50	NA	6.0	15.0	11
Ueno	P III	GEM + S-1 GEM S-1	68 66 68	NA NA NA	NA NA NA	15.9 12.7 13.8	12

^a^One-year progression-free survival.

It is possible that a better patient selection positively influenced the survival outcomes in the present study. However, previously published studies have shown similar survival outcomes by assessing FOLFIRINOX or GEM plus nab-paclitaxel regimens for LA or BRPC (Table 4)^14–17^. FOLFIRINOX has also shown a significant improvement of overall survival compared to GEM alone in metastatic pancreatic cancer^18^. Importantly, both in our study and in previously published data, a multimodal approach centring on potent systemic therapies was employed. Following the use of systemic chemotherapy, 40–80% of patients received CRT and 20–40% underwent surgical resection (Table 4). Such data indicate that multimodal treatment using these novel regimens provide significant survival benefits (median overall survival, 20–30 months). Limited evidence supports this strategy and the clinical significance of CRT is still controversial. However, we believe that the improved survival outcomes warrant further investigation.

**Table 4:** Published studies assessing GEM plus nab-paclitaxel or FOLFIRINOX for LA or BRPC. Abbreviations: FOLFIRINOX, fluorouracil, leucovorin, irinotecan and oxaliplatin; GEM, gemcitabine; PAXG, GEM, nab-paclitaxel, capecitabine and cisplatin; BR, borderline resectable; LA, locally advanced; CRT, chemoradiotherapy; PFS, progression-free survival; MST, median survival time.

Author	Design	Treatment	n	BR/LA	CRT	R0/1 resection	PFS (months)	MST (months)	ref.
Suker	Meta-analysis	FOLFIRINOX	315	0/315	64%^a^	26%^b^	15.0	24.2	15
Stein	Phase II	FOLFIRINOX	31	11/20	55%	42%	17.8	26.6	16
Gulhati	retrospective	GEM + nab-paclitaxel	99	45^c^/14/40	45%	15%	11.0^d^	18	14
Reni	Randomised phase II	PAXG GEM + nab-paclitaxel	26 28	10/16 15/13	88% 57%	31% 32%	12.5 9.9	20.7 19.1	17
Current study	retrospective	GEM + nab-paclitaxel	30	8/22	40%	20%	14.8	29.9	

^a^Pooled proportion of patients who received any radiation therapy in a random-effects model.

^b^Pooled proportion of patients who had resection in a random-effects model.

^c^Potentially resectable.

^d^Metastatic disease-free survival.

Objective response rate of GEM plus nab-paclitaxel (44.8%) was higher than conventional regimens for LAPC (5–41%) (Table 3). These results are consistent with those of the phase I/II study of GEM plus nab-paclitaxel for metastatic pancreatic cancer reported by Ueno *et al*. The authors showed that 69% of the patients (18 out of 26) underwent >30% shrinkage of pancreatic tumour^19^. In the era of neoadjuvant or conversion strategy for localised pancreatic cancer, first-line chemotherapy with higher response rate represents an attractive option.

In the present study, we found that first-line GEM plus nab-paclitaxel was well-tolerated and feasible in patients with LA or BRPC. The most common Grade 3 or 4 toxicity was neutropenia. However, patients rarely developed life-threatening infections or febrile neutropenia. The frequency of biliary tract infection (13%) was comparable to those observed in our previous prospective study (20%)^20^. The most common non-haematological toxicity was peripheral neuropathy in prospective studies^13,19^. In contrast, none of the patients developed grade 3 or more peripheral neuropathy in this study, possibly because the dosage of nab-paclitaxel was reduced or its administration was temporally discontinued when grade 2 peripheral neuropathy was observed.

The present study had some limitations. The main one is its retrospective nature. Thus, no standardized indication of pancreatectomy after induction of GEM plus nab-paclitaxel in BRPC was available. Furthermore, additional CRT was not performed according to predefined criteria. Radiographic evaluation was not performed according to the protocol specified duration. Consequently, objective response rate and PFS were biased. Finally, we enrolled a small number of patients, and the sample size was not calculated based on any statistical hypotheses.

The present study has an important fundamental strength. It included patients who started receiving first-line GEM plus nab-paclitaxel for LA or BRPC at our institution in a consecutive manner. We analysed all the treated patients, whether or not they received CRT or pancreatectomy based on an intention-to-treat basis. Furthermore, to avoid selection bias, we excluded from the study those patients who were referred from other institutions after the introduction of GEM plus nab-paclitaxel. Thus, the present study can provide precise data on survival outcomes of first-line GEM plus nab-paclitaxel for LA or BRPC in clinical practice.

In conclusion, our results demonstrate that first-line GEM plus nab-paclitaxel was well-tolerated and feasible in patients with LA or BRPC. Using a multimodal approach, we observed good survival outcomes (median survival of 29.9 months in all patients and 24.1 months in patients with LA). Although the study is limited by its retrospective nature with a small sample size, these results were better than those of patients treated with conventional therapies (e.g. upfront CRT or GEM-based chemotherapy). Further prospective studies are warranted to elucidate the effectiveness of first-line GEM plus nab-paclitaxel in a large cohort of patients.

## Materials and Methods

### Patients

We reviewed medical records of consecutive patients with PC who started chemotherapy at the division of Gastroenterology of Chiba Cancer Centre between January 2015 and August 2017. The following are the selection criteria of the study population: (1) Patients who started receiving first-line GEM plus nab-paclitaxel in our institution; (2) Pathologically confirmed pancreatic adenocarcinoma; (3) No prior chemotherapy or radiotherapy for PC; and (4) LA unresectable or BRPC, based on the NCCN clinical practice guidelines in oncology version 2.2018^21^.

Patients who were referred from other institutions after the introduction of GEM plus nab-paclitaxel were excluded to avoid selection bias.

### Treatment

Patients received intravenous nab-paclitaxel 125 mg/m^2^ followed by intravenous GEM 1000 mg/m^2^ on days 1, 8, and 15 every 4 weeks. According to the patient’s condition, GEM or nab-paclitaxel’s doses were modified at the physician’s discretion. Treatment was continued until the patient showed disease progression or unacceptable adverse events.

For patients who did not develop distant metastasis, we performed CRT using S-1 on the physician’s discretion following a minimum of a 2-week washout period of GEM plus nab-paclitaxel. Further contraindications for additional CRT included tumour invasion to the gastrointestinal tract, huge tumours, massive lymph adenopathy or ascites. We performed S-1-based CRT as reported previously^7,20^. In brief, a total dose of 50.4 Gy was delivered in 28 fractions. S-1 was administered twice a day on days 1 to 14 and 22 to 35. The daily S-1 dose was determined according to body surface area (BSA) as follows: BSA < 1.25 m^2^, 80 mg/day; 1.25 m^2^ ≤ BSA < 1.50 m^2^, 100 mg/day; and 1.50 m^2^ ≤ BSA, 120 mg/day. Maintenance chemotherapy was started after CRT until the patient showed disease progression or unacceptable adverse events, as reported previously^7,20^.

Indication for surgery was determined at the hepatobiliary pancreatic cancer board of Chiba Cancer Centre. Based on the radiographic findings and patient’s conditions, indication for surgery required consensus among radiologists, surgeons, pathologists and medical oncologists.

### Assessment

We evaluated the PS according to the ECOG (Eastern Cooperative Oncology Group) criteria. We assessed AEs according to the National Cancer Institute Common Terminology Criteria for Adverse Events, version 4. In the present study, we investigated AEs during first-line GEM plus nab-paclitaxel. We did not include AEs observed during maintenance chemotherapy after CRT. Radiographic responses were evaluated according to Response Evaluation Criteria in Solid Tumours, version 1.1.

### Statistical analysis

PFS was defined as the time between the date of treatment initiation and the date of disease progression or death. Overall survival was defined as the time between the date of treatment initiation and the date of death due to any cause. We used the Kaplan–Meier method to estimate the PFS and overall survival. The EZR ver. 1.35 software (https://cran.r-project.org/web/packages/RcmdrPlugin.EZR/index.html) was used to perform statistical analyses^22^.

### Ethics statement

The ethical review board of Chiba Cancer Centre approved the study. We performed the study in compliance with the 1964 Helsinki declaration and its later amendments and with the ethical guidelines for medical research by the Ministry of Health, Labour and Welfare of Japan. For this type of study (retrospective, non-invasive, observational study), written informed consent is not required. We used our institutional official website as an opt-out method.

## Data Availability

The datasets generated and/or analysed during the current study are available from the corresponding author on reasonable request.
